# Post-Partum Hæmorrhage

**Published:** 1882-05

**Authors:** 


### Post-Partum Hæmorrhage.

Dr. Barnes's article on post-partum hæmorrhage deals chiefly with the causes and treatment of the accident. We select for abstract that portion of it that relates to precautions against the dangers attending intra-uterine injections of perchloride of iron. To diminish the shock, the manœuvres should be as gentle as possible. The uterus should first be cleared of blood and clots with a stream of hot water; then, the organ being well compressed by the hand of an assistant, to reduce its volume and the extent of its inner surface, about a gill of the iron solution, at a temperature of about 80° F., should be slowly injected, taking care to secure it a free outlet by keeping the cervical canal well open. To lessen the danger of air entering the veins, in addition to compressing the uterus, all air should be expelled from the syringe before the latter is employed, and the nozzle should be inserted into the uterus very gently, taking special care as to holding the cervix open with the hand. The measures thus far mentioned are also the most trustworthy in guarding against a portion of the liquid being forced through the Fallopion tubes, as well as against the iron gaining entrance into the circulation and thus causing venous and cardiac thrombosis. To prevent embolism from decomposed clots in the uterine sinuses, besides the above-mentioned precautions, firm compression of the uterus should be maintained with a bandage, and ergotin should be injected subcutaneously soon after labor; quinine and ergot should be given, by the mouth or hypodermically, three times a day for at least fifteen days (a measure that the author has long made use of in all cases, whether iron was used or not), and the patient's nutrition should be kept up with the utmost care. To prevent septicæmia, from the decomposition of clots in the uterine vessels or in the cavity of the organ, the latter should be cleansed once or twice a day, after the first day, with carbolized water. For applying the iron solution the author has lately used large hard-rubber tubes, with large openings at the end. Pieces of sponge wet with the solution are stuffed into this end, and when the apparatus is in place the solution is squeezed out with a piston, thus causing such gentle contact of the liquid with the uterine surface that all shock is avoided. The injection, however, is the most convenient, and the author thinks a weak current of the solution the most efficient.—*N. Y. Med. Jour. and Obst. Rev.*

